# A scoping review of responsive caregiving in diverse populations and its association with child development

**DOI:** 10.1016/j.earlhumdev.2025.106424

**Published:** 2025-10-19

**Authors:** Eunice Lobo, Sandeep Mahapatra, Joshua Jeong, Giridhara Rathnaiah Babu, Prashanth Nuggehalli Srinivas, Debarati Mukherjee, Onno C.P. van Schayck

**Affiliations:** a Indian Institute of Public Health - Bengaluru, Public Health Foundation of India, Bengaluru, India; b PHFI Centre for Developmental and Lifecourse Research, Bengaluru, India; c Centre for Health Systems, Institute of Public Health Bengaluru, Bengaluru, India; d Department of Family Medicine, Care and Public Health Research Institute (CAPHRI), Maastricht University, P.O. Box 616, Maastricht, 6200, MD, the Netherlands; e Independent researcher, Bengaluru, India; f Hubert Department of Global Health, Rollins School of Public Health, Emory University, Atlanta, GA, 30322, USA; g Department of Population Medicine, College of Medicine, QU Health, Qatar University, P.O. Box: 2713, Doha, Qatar

**Keywords:** Caregiver-child interactions, Maternal sensitivity, Responsiveness, Nurturing care, Global south, Early childhood development

## Abstract

**Background:**

Responsive caregiving is a key component of nurturing care and crucial for early child development. While responsive caregiving has been examined in multiple studies, a comprehensive review summarizing findings from these studies across diverse caregiver, child, cultural, and socio-economic contexts is currently lacking.

**Methods:**

We conducted a scoping review to synthesize evidence on (1) caregiver, child, and contextual factors influencing responsive caregiving and (2) association between responsive caregiving and children’s neuro-development and mental health. We included peer-reviewed English articles describing responsive caregiving for children aged 0–8 years. Articles were systematically searched in PubMed, Web of Science, APA PsychInfo, APA PsycArticles, SocINDEX, Google Scholar, and the reference lists of included articles. Relevant data were extracted, collated, and synthesized into descriptive summaries and associations with children’s development.

**Results:**

We retrieved 7412 unique studies for title/abstract screening and 541 full-texts were screened and 138 studies met the inclusion criteria. Caregiver characteristics, including caregiver type, maternal health, and demographics, influenced responsive caregiving, with notable differences between mothers and fathers. Child-level factors, such as developmental disabilities, age, term or preterm birth status, and gender, also shaped responsive caregiving. Cultural context and socio-economic status also influenced responsive caregiving across populations. Language development was the most frequently reported with responsive caregiving.

**Conclusion:**

This scoping review maps how caregiver, child, and contextual factors influence responsive caregiving. Significant gaps remain in understanding caregiving in the under-researched Global South settings. Policymakers and practitioners should consider socio-cultural contexts, along with the pathways and mechanisms, when designing inclusive interventions that strengthen caregiving and support child development.

## 1 Background

The Nurturing Care Framework (NCF) for early childhood development [1], released in 2018 by the World Health Organization (WHO), UNICEF, and other developmental agencies, comprises five key components that promote children’s development. These are: good health, adequate nutrition, responsive caregiving, safety and security, and opportunities for early learning. Responsive caregiving is a critical component of the NCF and refers to the extent to which caregivers are attuned to their child’s emotional state, aware of their child’s cues, and can respond in a prompt, contingent, and developmentally-appropriate manner. [2,3]. It encompasses “serve-and-return” interactions - the back-and-forth exchanges in which caregivers notice, interpret, and sensitively respond to children’s signals and needs. [4]. To guide our review, we adopted the definition of responsive caregiving presented by Eshel et al. (2006) [5], and further refined by Black and Aboud (2011) [6]. This definition emphasizes caregiver responses that are prompt, contingent, and developmentally appropriate to children’s cues. It also highlights the dynamic, reciprocal nature of caregiver-child interactions, where children’s signals, behaviours, and developmental needs shape caregiving responses over time. Therefore, this review focuses on three interrelated dimensions of responsive caregiving. First, it examines caregiver behaviours that are contingent upon the child’s cues, prompts, sensitivity, and developmentally appropriateness. Second, it considers the child’s abilities and developmental needs, emphasizing the reciprocal and dynamic nature of caregiver–child interactions, with the child as an active participant in the caregiving process. It underscores the importance of individualized approaches to support optimal development. Third, the review addresses the broader contexts in which caregiving occurs, including cultural settings (such as individualist versus collectivist societies), socioeconomic conditions, and urban–rural differences. This framing aligns with broader theoretical frameworks of parenting that highlight the interplay between caregiver factors (e.g., mental and physical health), child factors (e.g., temperament, developmental status), and contextual stressors or supports (such as poverty, social support, cultural norms) [7,8]. Thus, illustrating how both the child and the caregiver influence each other over time.

Recent research has demonstrated that children’s brain development is closely related to responsive caregiving, particularly during the critical early childhood period. Responsive caregiving has been shown to improve children’s cognitive, language, and social-emotional development [9,10] and improve adolescent and adult human capital production [11,12]. However, a recent scoping review on the measurement of nurturing care practices [13] demonstrated that while the majority of the 239 studies reported on nutrition, early learning, and health measures, only 78 reported measures of responsive caregiving.

Furthermore, differences in caregiving norms, parenting practices, and parent-child interactions have been observed between the Global South and the Global North, as well as among various socio-demographic factors [14]. Two recent studies showed that family poverty can negatively shape parenting practices, leading to less warmth, less supportive interactions, and a greater tendency towards harsher or less consistent parenting approaches, which in turn impedes children’s cognitive and overall development [15,16]. Responsive caregiving has also been shown to vary by parental factors (e.g., age, mental health, social support, beliefs about child-rearing). Child-related characteristics, such as developmental delays, intellectual disabilities, or preterm birth, can also influence the quality of responsive caregiving received. These variations may emerge out of caregiver-related challenges (e.g., increased stress, limited social support, financial burden) and the nature of the child’s developmental condition, which may involve subtler or difficult-to-interpret cues and thereby require higher responsive caregiving [17–19]. These findings emphasize the importance of studying responsive caregiving in diverse populations and contexts to gain a more nuanced understanding of its influence on children’s development across diverse global settings [20–26]. However, a comprehensive review of the existing literature on responsive caregiving, which includes studies from low-income rural settings, especially in the Global South, is lacking.

While the importance of responsive caregiving is widely acknowledged, previous reviews have noted inconsistent conceptualization and limited use of standardized measures. For example, Jeong et al. [13] found that only one-third of studies clearly included responsive caregiving constructs, and that such constructs were often combined with early learning and not described as a distinct construct. There is little consensus on how it should be defined or operationalized across contexts [27]. This scoping review aims to bridge this gap by summarizing the factors that influence responsive caregiving across global settings. This is essential for developing contextually tailored and culturally sensitive interventions and policies for child development, optimizing children’s development across global settings, and enhancing their acceptability and feasibility within specific populations. This review also highlights the contexts and settings where research on responsive caregiving is currently limited, informing where future funding opportunities should be focused to improve the global representativeness of the findings.

## 2 Research questions

The following research questions guide this review:

1. What caregiver and child factors (e.g., health, demographic, developmental status, culture, etc.) influence responsive caregiving?
2. How is responsive caregiving associated with child neuro-development and mental health?

The specific objectives of this review were to identify and map the literature on responsive caregiving across global settings and provide a narrative summary of responsive caregiving categorized by attributes related to caregivers, children, households, culture/communities, and location (urban/rural). Given the broad and exploratory nature of our research questions, a scoping review was considered appropriate for summarizing the findings. The synthesis of responsive caregiving across diverse global settings and contexts will help inform early childhood development policies and interventions tailored to diverse settings, ultimately supporting children’s development worldwide.

## 3 Methods

This scoping review was conducted following the Arksey and O’Malley framework [28] and the Joanna Briggs Institute (JBI) methodology for scoping reviews [29]. We present our findings according to the Preferred Reporting Items for Systematic Reviews and Meta-Analyses (PRISMA) guidelines for scoping review (PRISMA-ScR) [30] (Supplementary file 1).

### 3.1 Eligibility criteria

The inclusion criteria were: (i) primary peer-reviewed articles published in the English language from January 1982 to September 2024 (timeframe chosen to capture the evolution of responsive caregiving over the last four decades); (ii) described responsive caregiving between any caregiver and a child aged 0–8 years. Using the definitions by Eshel et al. [5] and Black & Aboud [6], we operationalized responsive caregiving as three interrelated dimensions: caregivers noticing and interpreting the child’s signals and cues, caregiver’s sensitivity and emotionally supportive behaviour towards the child, and contingent and prompt responses to the child’s cues. Studies that reported the association of responsive caregiving with child neurodevelopment and mental health were also included, but reporting child development measures was not essential for inclusion. Articles using quantitative and/or qualitative methods were included. Editorials, commentaries, reviews, and grey literature were excluded.

### 3.2 Search strategy and data sources

The search string was prepared using relevant keywords, synonyms, related terms, and medical subject headings (MeSH terms) aligned with the inclusion criteria described above (as an example, see Supplementary file 2, which lists the search strategy used in PubMed). The first author (EL) consulted with senior authors (DM, PNS, JJ) and reviewed similar topics to gather relevant terms. Boolean operators like “AND,” “OR” and “NOT” were used to connect and refine the search string. PubMed-related search modifiers such as phrase searching, truncation, field tags, and filters were included. Once the search string draft was ready, a medical librarian at Maastricht University was consulted to refine and finalize the search strategy for PubMed. The first author adapted the final version of the PubMed search string for the remaining databases - Web of Science, APA PsychInfo, APA PsycArticles, and SocINDEX. In addition, Google Scholar was manually searched using relevant keywords to capture additional articles not indexed in the selected databases. The citations of all included full-text articles were manually checked to ensure the inclusion of eligible articles not returned by the above search attempts.

### 3.3 Reference management

All articles retrieved from the electronic database searches were imported into the web-based bibliographic manager, Rayyan [31] and duplicates were removed. A two-stage screening process was used to assess the relevance of studies identified in the search. Two authors (EL, SM) independently screened all articles for title and abstract relevance in Rayyan.ai in blind mode. They met weekly to discuss and resolve conflicts and clarify uncertainties related to study selection [32]. Cohen’s kappa statistic [33] was chosen to assess inter-rater agreement. The overall kappa for the title/abstract screening was 0.91. After title/abstract screening, all the included articles were downloaded for a full-text review. Only articles with available full texts were included. Relevance was assessed by both reviewers (EL, SM) using the inclusion/exclusion criteria defined above.

### 3.4 Data extraction

Two authors (EL and SM) developed and piloted a form in Microsoft Excel to extract relevant study characteristics from the final list of included full-text articles. The data extraction form was piloted using 10 randomly selected articles, which were double-coded by EL and SM to assess the consistency of data extraction, and checked for any ambiguities or areas that required refinement to improve the clarity and comprehensiveness of the form. The pilot was completed once both reviewers agreed on the final version of the extraction form. The senior authors then reviewed this form. Iterative changes were made based on the feedback received. Data extraction commenced with the final version of the form and was completed by EL and SM. Data extraction fields included author, title, publication year, objective or research question, location, study design and tools, study participants’ characteristics (age, sex, education, occupation, income status), study findings relevant to our research questions (caregiver, child, socio-economic status, & cultural factors impacting responsive caregiving, and child development). We also documented the construct(s) of responsive caregiving as reported by the authors in each study, and the methods for their measurement. The remaining articles were single-coded, with regular discussions to resolve discrepancies and standardize coding. Regular meetings with senior authors were conducted during the data extraction process to address questions, clarify uncertainties, and monitor progress.

### 3.5 Quality appraisal

EL and SM also conducted a quality appraisal of the studies using the JBI Critical Appraisal Checklists for each type of study [34]. Each study was independently assessed according to the relevant checklist criteria, and the findings were compiled and analysed to identify methodological strengths and weaknesses of all included studies. Each study was categorized as low, medium, or high quality based on the criteria laid down by the JBI checklists (high = ≥80 % of checklist items satisfied; medium = 50–79 % satisfied; low ≤50 % satisfied).

### 3.6 Data analysis and presentation

Data extracted from the included articles were summarized using two main methods: (i) descriptive statistics to report the frequency and percentages of articles reporting various caregiver and child characteristics (age, sex, type of caregiver, caregiver education, caregiver occupation, income status, etc.); and (ii) a narrative summary of responsive caregiving across the above categories and its associations with child development. For qualitative studies, the results and quotations included in the articles were analysed, and relevant data were presented as a narrative summary.

### 3.7 Ethical considerations and study protocol

Ethics approval was exempt for this study, as the analyses and reporting of results were based on data retrieved from publicly available data in published studies. The study protocol has been published else-where [35].

## 4 Results

Description of the included studies and study sample

**Fig. 1:**
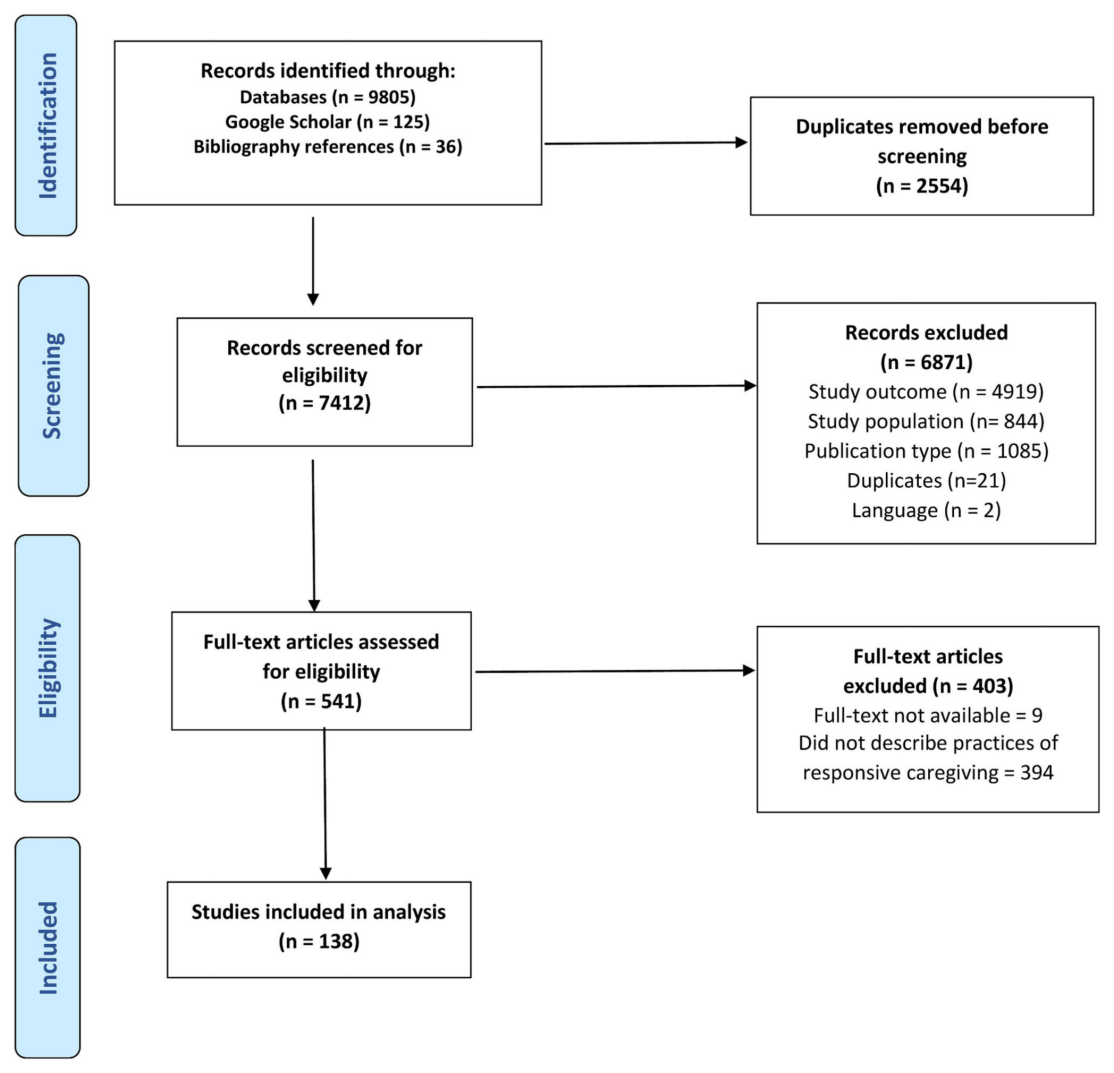
PRISMA flow diagram of search process and outcomes.

A total of 9805 articles were identified from the targeted electronic database searches. Manual searches in Google Scholar identified an additional 125 articles, and 36 articles were included from the bibliographies of the included full texts. After removing 2554 duplicates, the titles and abstracts of 7412 articles were screened. 541 articles met criteria for full-text screening, and finally 138 articles were included in the scoping review (see PRISMA flowchart, Fig. 1).

As seen in Table 1, of the 138 studies, 133 used quantitative study methods, one used qualitative enquiry, and four were mixed-methods studies. Among the quantitative studies, 61 % (*n* = 81/133) were cross-sectional, 31 % (*n* = 41/133) were longitudinal or cohort studies, 7.5 % (*n* = 10/133) were randomized controlled trials, and one was a case-control study. Most articles (82 %; *n* = 113/138) were published in the past two decades (2003 onwards), and 63 % (*n* = 87/138) had a sample size of less than 100. Studies were predominantly from the Global North, specifically the Americas (57 %; *n* = 81) and Europe (30 %; *n* = 42), while only 9 % studies (*n* = 12) were from Africa, and 4 % (*n* = 6) were from South-East Asia. Some studies were conducted in more than one region; therefore, the total percentage exceeds 100 %.

While all studies recruited mothers as the primary caregiver, 28 studies also included other caregivers such as fathers, grandmothers, aunts, and siblings. One study included day-care providers as caregivers, along with mothers. The median parental age was 33 years among studies that reported caregiver age. The age range for children varied from 3 weeks to 8 years, with an almost equal proportion of boys and girls.

Brief overview of the measurement tools and constructs used across studies

Although evaluating measurement tools was not a primary objective of this review, we briefly summarize the tools used across studies to assess responsive caregiving. A variety of tools and approaches were used (see Table 2). The predominant methods involved videotaped mother or caregiver–child interactions (*n* = 103). These included home-based play or laboratory-based observations (free play and/or structured tasks). Most studies used standardized or validated coding tools (*n* = 59), while others adapted coding schemes from previous studies (*n* = 12), and some did not specify the coding scheme/tool used (*n* = 32). Specific subscales measuring responsive caregiving within standardized assessment tools, such as the Home Observation for Measurement of the Environment (HOME) Inventory (*n* = 18) and the Nursing Child Assessment Teaching Scale (NCATS) (*n* = 8), were also commonly used. Tools like HOME and NCATS are multidimensional and include assessments of the early-learning environment, along with responsive caregiving. Furthermore, a diverse set of standardized questionnaires and observational coding systems was employed (e.g., Ainsworth Sensitivity Scale, Emotional Availability Scales), which typically assessed caregiver-child interactions across various domains, including maternal responsiveness and sensitivity. Some unique instruments (PICCOLO, Social Interaction Measure for Parents and Infants, Thorpe Interaction Measure) appeared in a few studies, highlighting ongoing methodological innovations. The methodological details of the individual studies are presented in Table 2, and Supplementary Fig. 1 presents the measurement tools used across studies.

The predominant construct measured was responsivity (such as responsiveness, verbal responsiveness, etc.) in 75 studies (54.3 %). The second most frequently assessed construct was sensitivity (including sensitiveness, sensitivity to cues), which was measured in 57 studies (41.3 %). Contingency (including contingent response) was the assessed in 14 studies (10 %), while positive affect (including positive regard) was assessed in 13 studies (9.4 %), and compound terms (such as sensitive responsiveness) in seven studies (5 %). It is important to note that many studies assessed multiple constructs, hence percentages do not add up to 100 % (Table 2).

Risk of bias of the included studies

Critical appraisal of the included studies revealed a predominance of high-quality studies (79 %; *n* = 109/138), with rigorous methodologies, including clear inclusion/exclusion criteria, and appropriate statistical analyses (Table 2).

### 4.1 Responsive caregiving in relation to caregiver factors

#### 4.1.1 Caregiver physical and mental health (n = 28)

Regarding physical health, a study comparing mothers living with and without HIV found that both groups exhibited similarly low levels of sensitivity in southeastern U.S. [36]. While mothers with iron-deficiency anemia exhibited low responsiveness towards their infants during the first year of life [37].

Several studies reported that mothers with mental health issues, including those with childhood-onset depression [38], post-traumatic stress disorder [39], postnatal depression along with suicidal tendencies [40], high trait anxiety [41], unipolar and bipolar depression [42–49], antenatal or postnatal depression [50,51] reported as less sensitive and responsive towards their children, compared to mothers without any mental health conditions. In fact, Gerstein et al. reported the long-lasting effects of poor maternal health, showing that even when preterm-born children were 5 years of age, mothers who had reported higher levels of depression at the time of neonatal intensive care unit (NICU) discharge were less sensitive compared to mothers with lower depression [46].

Among intervention studies, two studies that focused on improving mother-child interactions and responsive caregiving demonstrated improved sensitivity and responsive caregiving post intervention [52,53]. These observations were supported by a study that showed that parents with high psychological well-being were more sensitive to their children as compared to those with low psychological well-being [54]. On the contrary, mothers who used drugs during pregnancy or were undergoing treatment for substance abuse (e.g., cocaine, heroin, opioid, methadone, or buprenorphine) showed lower levels of sensitivity and contingency towards their children compared to control mothers [55–58].

Evidence from this review also suggests that maternal sensitivity is shaped by the broader social and psychological environments, as seen in studies by Murray et al. (1996, 2003) that reported mothers experiencing lower levels of social adversity exhibited higher sensitivity towards their children in comparison to well women [44,52]. Similarly, a study conducted in the slums of Yemen found that greater social support was associated with more sensitive mother-child interactions [59], while in rural Mexico, adolescent mothers identified their mothers-in-law and partners as important sources of support, which contributed positively to their caregiving [60]. In contrast, a study from the slums of Makassar in Indonesia showed that mothers with a history of childhood maltreatment exhibited lower levels of sensitivity in interactions with their children relative to mothers without such histories [61].

#### 4.1.2 Caregiver demographics (n = 15)

Videotaped observations of mother-child interactions showed that adolescent or younger mothers exhibited lower responsivity or sensitivity, paid less attention, and spent less time looking at their child compared to older mothers. Reasons attributed to this included poor executive functioning in teen mothers, socio-economic disadvantage, higher rates of depressive symptoms, and low social acceptance [43,60,62–67]. However, an intervention study involving adolescent mothers in Australia demonstrated that adequate support, information and help to teenage mothers can significantly improve maternal sensitivity [68]. Maternal intelligent quotient also positively influenced maternal responsivity towards children as noted in a study by Sterling et al. [69]. Working mothers were found to be more verbal and sensitive to their children, compared to non-working mothers [54,70]. Further, parents with higher education [49,50,54,71] and married parents, especially those with high marital quality (assessed using the Quality Marriage Index, and shortened version of the Spouse Observation Checklist) were more sensitive to their children compared to the control groups [54,71].

#### 4.1.3 Caregiver relation to child (n = 13)

Among studies that compared responsive caregiving among mothers and fathers, two studies showed that mothers provided more sensitive care to children compared to fathers [71,72], but others also showed comparable responsiveness by both parents [73–75]. However, mothers were better in noticing and responding to children’s emotional cues among those born preterm or with neurodevelopmental delays, where the cues elicited are often weaker or more difficult to understand by caregivers [73,74,76].

In an intervention study that compared paternal responsiveness to children’s distress cues, no differences were noted between the lower or higher involvement intervention arms [77]. No difference in responsivity was also observed during play or mealtimes in another study comparing primary (who primarily cared for their infant) and non-primary fathers [78].

Three studies on responsive caregiving of biological versus adoptive caregivers in the Global North contexts found no differences in overall sensitivity or responsiveness to the child’s cues and non-distress signals [79,80], suggesting that biological relationships may have little influence on caregiving.

Two studies examining responsive caregiving in mothers of children who were naturally conceived versus those who were conceived through medical assistance showed no difference in responsiveness to children’s cues [81]. Responsive caregiving among mothers who conceived through artificial insemination appeared to vary by donor type, with those who received insemination from their married partners showed higher levels of responsiveness to their children compared to those from external donors [82].

One study comparing responsive caregiving between mothers and daycare providers demonstrated that mothers provided more contingent responsiveness [83].

#### 4.1.4 Caregiver feeding practices (n = 11)

Mothers who breastfed their infants were more responsive and sensitive to infant cues compared to mothers who bottle-fed their infants [84,85]. Similarly, mothers from rural Gusii in Kenya, and Andean and Amazonian parts of Peru, practiced sensitive responsiveness by breast-feeding their children after observing their children’s glances, or cues [86,87]. While, mothers who used screens/mobile phones during feeding showed lower verbal encouragement compared to mothers who did not use any device/screens [88].

Five studies (including one intervention from Bangladesh that trained mothers on responsive caregiving) reported that higher levels of caregiver encouragement and sensitivity led to children more likely to enjoy food, have more mouthfuls of food, and signal for more food [89–94].

### 4.2 Responsive caregiving in relation to child factors

#### 4.2.1 Child developmental status (n = 27)

Five studies reported responsive caregiving of mothers with atypically developing children. Mothers showed high levels of responsive caregiving across structured and unstructured tasks due to the child’s language skills and development status [69,95–97]. Compared to mothers of children with intellectual disabilities or developmental delays, those having children with physical impairments used significantly more proximal gestures such as touching, holding, and kissing as responsive caregiving [98].

Twelve studies comparing responsive caregiving of mothers with mentally- or physically impaired children versus those with typically developing children found differences in both the *level* and *type* of responsive caregiving among these groups. Lorang et al. showed that both groups (mothers of typically and atypically developing children) could recognize the gestures, cues, and behavioural signals of their children equally well [99]. In general, the level of responsive caregiving was higher in mothers with typically developing children [100–105]. A Canadian study investigated mothers’ interaction with children before and after a disability diagnosis. They found that the levels of contingency (touching, talking, smiling, looking) decreased right after diagnosis (when children were 6–9 months old), but reverted to comparable levels as mothers with typically developing children by 12 months of age [106]. While mothers of children with familial risk for Attention Deficit Hyperactivity Disorder (ADHD) in an Israeli study showed low levels of responsiveness compared to mothers with typically developing children, fathers showed no such difference [107].

In terms of the *type* of responsive caregiving, mothers of atypically developing children were more likely to use gestures and supportive behaviour [108,109], while mothers of typically developing children used higher levels of verbalizations [110]. This indicates that caregivers tailor responsive caregiving to specific developmental challenges among children, particularly in cases of intellectual challenges, where children may exhibit communication cues that may be more difficult to understand compared to children who are typically developing, or those with physical impairments.

We also identified 10 studies that compared responsive caregiving in term versus preterm-born children. Similar to observations of higher levels of responsive caregiving for typically developing children, these studies also reported higher levels of responsive caregiving for term-born versus preterm children [111–113]. These behaviours were not restricted to the infant stage, but extended to when children were older. For example, Erickson et al. [114] showed that preschool-aged children born preterm received less responsivity compared to their full-term born peers. In the same vein, studies comparing premature twins versus singletons found that mothers of premature twins demonstrated lower responsiveness and exhibited fewer instances of holding, touching, talking, or patting their child, compared to mothers of premature singletons [115,116]. However, some studies reported contrasting results or no differences between groups [117–120], potentially suggestive of context-specific differences and requiring more in-depth and nuanced studies to unravel this relationship.

#### 4.2.2 Child demographics (n = 20)

Caregivers typically adjusted their responsive caregiving as children grew up, shifting from physical displays of affection with infants, to more vocal forms of engagement as children developed language and motor skills [121]. In longitudinal observations, a decline in contingent responding was noted from 4 weeks in a Cameroon sample, whereas similar declines were observed around 12 weeks in samples from Germany and Denmark [122,123]. Mothers of older children in Finland showed higher levels of sensitivity compared with mothers of younger children [50], a pattern consistent with Bornstein et al.’s finding that, as typically developing children age, mothers increase imitations and expansions [124]. Similarly, Sterling et al. [125] also reported increases in maternal responsivity with age among atypically developing children, particularly in facilitative gestures. It is worth noting that some but not all studies suggested that the level of contingent responses changed as children grew older, but the direction and rate of change varied across contexts. Fathers in one study showed reduced responsivity as sons grew older, a pattern not observed for maternal responsiveness to girls [71,117]. Two studies reported that mothers who showed high levels of responsiveness in infancy, continued to do so across the preschool years [126,127]. In contrast, studies from Spain, United States, and South Korea demonstrated that caregivers’ sensitive and contingent responses *increased* longitudinally with infant age [128–130].

Eight studies reported parental interactions based on the gender of the child. A study from the United States found that employed mothers were more attentive to their daughters as compared to non-employed mothers to their sons [70], while a cross-country comparison study (Argentina, USA, Italy) showed that mothers were more sensitive to their daughters than to their sons [131]. A study also noted an interaction between age and gender, reporting that as infants turned one year of age, the frequency of maternal responsiveness increased only for girls in comparison to boys [132]. Mothers with childhood-onset depression were also more responsive to their daughters than their sons [38]. Other studies found either no consistent gender difference or contrasting patterns such as, higher responsiveness to sons as compared to daughters, among adolescent mothers, adoptive mothers, or mothers of preterm infants [67,80,133]. During a free-play task, one study also found that fathers were more responsive to their sons compared to daughters [76].

#### 4.2.3 Child health (n = 3)

Responsive caregiving is a reciprocal process in which children’s signals elicit caregivers’ responses, which shape subsequent child signaling and engagement. Mothers of children with poor health or developmental status showed reduced responsiveness as compared with mothers of healthy or typically developing children, pointing to a potential impact of children’s health status on caregiver engagement. A study from the United States involving infants [134], and another from Costa Rica of 5-year-old children [135] both showed lower responsivity and contingent behaviour when caring for children with iron-deficient anemia, compared to children with normal iron/hemoglobin levels. Similar observations were reported by a study on feeding styles and mother-child interactions among stunted children in rural Ethiopia, which showed that mothers had poor feeding style, breast-feeding practices, and were also less responsive to their child’s cues [92].

### 4.3 Responsive caregiving in relation to context

#### 4.3.1 Cultural/location differences (n = 16)

Responsive caregiving showed considerable variation across cultural contexts. In Global North cultures, responsive caregiving was often characterized by verbal engagement, while Global South settings emphasized higher levels of physical proximity and non-verbal interaction styles [131,136]. Two studies by Bornstein et al. [137,138] showed that mothers in the U.S. and France engaged with children through verbal prompts and showing objects, while mothers from Japan interacted more frequently through touch, kissing, hugging, and looking at their children. Similarly, comparative studies between mothers from Muenster in Germany and mothers from Cameroon and Kenya [86,122,139] showed that mothers from Muenster used higher levels of visual and auditory responsiveness through verbalizations, vocalizations, and visual cues. Further, Canadian mothers responded more to their children’s utterances with imitations or expansions, while native Italian mothers used more utterances [140].

Differences in urban and rural contexts played a significant role in the way mothers responded to their children. Studies reported urban/rural differences in caregiving, with urban caregivers tending to exhibit more responsivity than their rural counterparts, with the latter having limited time due to household and agricultural chores. As seen in the studies with mothers from urban San Francisco (USA) and urban Iran were more responsive towards their children as compared to mothers from rural Pacific Northwest, and rural Iran [141,142]. Mothers from rural Iran, Fiji, and Kenya were more likely to respond to negative expressions, negative bids, or vocalizations by the child [142,143]. Similarly in Yemen and Costa Rica [59,144], low levels of sensitivity were noted towards children, with mothers more focused on their chores, and faced difficulties in responding to children’s non-distress cues. In rural Peru and Malawi, mothers reported little to no time to tend to their child due to household and agricultural chores [87,145]. This prompted other caregivers (grandparents, fathers, etc.) to gradually increase their contribution to childcare as children grew older. Among the Mbendjele hunter-gatherers of the Republic of Congo, caregivers responded quickly to infant crying with comforting or feeding, with non-maternal caregivers (fathers, siblings, grandparents, and more distant relatives) also providing substantial levels of care for the child [146]. We also noted that while caregivers in Malawi expressed unfamiliarity with the concept of “responsiveness,” observational data showed that their interactions were indeed responsive, though often non-verbal in nature [145].

#### 4.3.2 Socio-economic status (n = 8)

Overall, responsive caregiving was significantly affected by socio-economic constraints, as is expected. Caregivers from low-income backgrounds often exhibited low levels of responsive caregiving due to financial and social stressors, which was especially evident in cases of intervention studies that trained caregivers on responsive caregiving, but mothers reported lacking the means or resources to fully implement the recommendations [145]. Four studies from the United States [71,111,147,148] and studies from low-income urban slums in Makassar, Indonesia, and Rio de Janeiro, Brazil [61,149] all reported that caregivers from low-income backgrounds showed low levels of sensitivity. A Pakistani study in a rural setting confirmed these observations by reporting lower levels of responsive caregiving behaviours among those belonging to the lower socio-economic group in comparison to higher socio-economic groups [49].

Overall, these studies highlight the essence that caregiving practices are shaped by local cultural norms, household structures, and contextual factors.

### 4.4 Association between responsive caregiving and child development (*n* = 38)

Given the exploratory scope of this scoping review and the pre-dominance of cross-sectional studies, we report only associations and not causal interpretations. Many studies reported positive associations between responsive caregiving and child development, particularly in language, cognitive, and socio-emotional domains. Responsiveness was associated with children reaching language milestones earlier, better cognitive performance, and positive socio-emotional domains, in both typically developing children and those with developmental delays or health conditions. However, a few studies reported no observed effects, suggesting that the impact of responsive caregiving on development may vary depending on the context, measurement methods, or specific developmental domain. Overall, these findings highlight the vital role of responsive caregiving in promoting child well-being, with consistent and robust evidence supporting responsive caregiving as a key contributor to positive development.

Studies found that responsive questions, utterances, labels, were significantly correlated with better language development [94,132,140,150–154] and the timing of children reaching their language development milestones [155–157]. For atypically developing children, parents using more follow-in language with an orienting cue led to significant improvements in spoken vocabulary [158–160]. Additionally, in a study involving children with autism spectrum disorders, responsive communication by fathers was also positively associated with children’s language scores [73].

The six studies investigating development related to intelligence, cognition, motor, and literacy skills, including among children born preterm, reported that high and consistent maternal responsiveness played an important role in increasing cognitive and literacy skills during early childhood [114,126,127,161–163]. In typically developing children, parental sensitivity was also associated with higher intelligence, larger vocabularies, and cognitive scores [45,71,164,165]. Two studies reported improvement in cognitive and language skills associated with increased caregiver responsivity [65,166]. Additionally, similar findings were observed in intervention studies focused on maternal support & promoting parenting, child development, and book sharing & responsive feeding, respectively - all of which showed improved cognitive and mental development scores [58,167–169].

Further, high levels of responsive caregiving was associated with positive socio-emotional domain for typically developing children from the Global North and South alike (Pakistan, South Africa, Peru, United Kingdom) [49,165,167,170]. Among preterm-born children in Canada, lower internalizing behaviours were noted for children with caregivers showing higher sensitivity as compared to term-born children [171]. On the other hand, a moderate association was observed between children’s playfulness (a socio-emotional characteristic) and parental responsiveness among children with motor delays [74].

Further, some studies found no differences in child development as a function of responsive caregiving. In Tanzania, variations in emotional and verbal responsivity by caregivers were not associated with children’s language, cognitive, and motor skills at 15-months [172]. A Chinese study also found that maternal responsivity measured at 9 months did not have any effect on children’s executive functioning skills at 2–3 years of age [173].

## 5 Discussion

This scoping review provides a comprehensive synthesis of responsive caregiving worldwide, and documents variations based on caregiver factors, children’s individual needs, abilities, and developmental stages, as well as the attributes of the study setting and population, such as income level, cultural nuances, and geographical location. There is increasing recognition of the pivotal significance of the early childhood period in shaping lifelong health and productivity. The *Lancet* series on Early Childhood Development (2007, 2011, 2016) [174–176], followed by the launch of the Nurturing Care Framework in 2018 [1], highlighted responsive caregiving as a cornerstone for promoting children’s development. These seminal reports have spurred heightened interest in research and interventions in this area; therefore, it is unsurprising that the majority of the articles included in this review were published in the past two decades. While research on responsive caregiving has grown, our review showed that studies are predominantly from the Global North. Although the Global South houses the majority of the world’s children, the limited coverage from this region preempts efforts to develop a truly global understanding of diverse responsive caregiving and its determinants. Future research funding and capacity-building efforts must focus on being more inclusive and geared towards under-represented regions and populations.

### Variations in responsive caregiving in relation to caregiver factors

Our review highlighted that poor maternal health, especially maternal *mental health*, compromises the quality of caregiver-child interactions, as mothers become emotionally unavailable and withdrawn, and less sensitive to their children’s cues and behaviours [177,178]. Furthermore, while adolescent mothers exhibited lower responsiveness due to various socio-economic and psychological factors, targeted interventions can improve their caregiving abilities. These findings echo the Nurturing Care Framework’s emphasis on the need to *‘care for the caregivers’*, and emphasize the need to strengthen platforms that support nurturing care among vulnerable caregiver populations.

Although considerably limited in focus and coverage, emerging literature has begun to highlight the seminal role of father’s involvement in children’s social, motor, and language development. Only two studies focused exclusively on fathers as caregivers, with most studies making comparisons between the responsive caregiving of fathers versus mothers, including children with developmental delays and disabilities. Similar to Bentenuto et al. [179], our review noted that increased interactions between fathers and children with autism spectrum disorder improved children’s language scores. Future studies should focus on developing a more nuanced understanding of the nature of fathers’ involvement in childcare across diverse cultural settings, and build on the limited evidence to inform interventions that engage fathers more effectively in early childhood care. Promoting men’s engagement in childcare will not only bolster more equitable and high-quality responsive care, but also reduce the exclusive burden of caregiving on mothers [180,181].

### Variations in responsive caregiving in relation to child factors

The caregiver-child relationship is reciprocal in nature [182,183], wherein both caregiver and child characteristics mutually influence the dyadic relationship. In the context of children with developmental delays or disabilities, responsive care is influenced by their level of functioning, since maternal responsiveness is likely dependent on how clearly a child is able to communicate (verbally or otherwise) their needs and wants. Children with physical impairments tend to receive more responsive and sensitive care compared to those with intellectual impairments, perhaps due to the more visible nature of the impairment and heightened vigilance to ensure safety and security against potential harm in the former, and weak or subtle behavioural cues elicited by the child in the latter [184,185]. Furthermore, longitudinal studies on responsive caregiving as children grew older highlight the dynamic nature of responsive caregiving. Children acquire new capabilities with age, expanding the scope of their demands for care and attention [130]. Studies demonstrated that caregivers adapt to children’s growing abilities and emerging needs by modifying their caregiving to ensure that interactions remain age appropriate.

Caregivers of preterm infants exhibited lower levels of responsive caregiving as compared to caregivers of term born infants. This finding suggests that additional underlying factors, such as maternal stress, exhaustion, and the unique demands of caring for premature infants or twins, can potentially impact the quality of responsive caregiving [186,187]. However, we must also be cognizant of the reciprocal nature of responsive caregiving and note that preterm infants frequently exhibit subtler socio-emotional cues, which can be challenging for caregivers to interpret, thereby impacting their ability to provide responsive and sensitive care. Therefore, caregivers with small and vulnerable infants, or children with developmental delays or disabilities, should receive specialized attention and tailored resources and interventions to enhance responsive caregiving, including the recognition and interpretation of weak or subtle cues elicited by vulnerable babies, and support for providing prompt caregiving responses that are appropriate for the child’s developmental status. This is likely to promote positive child developmental trajectories in this population [188–190].

Gender-based differences in responsive caregiving were observed, with some caregivers being more attentive and responsive to daughters, and others to sons. These findings suggest that culturally ingrained gender norms and attitudes may significantly impact the quality of caregiver-child interactions. Given these inherent biases in certain populations, future interventions should make caregivers aware of these biases and include components that motivate them to engage equitably with children regardless of their gender.

### Variations in responsive caregiving in relation to cultural and socio-economic contexts

Cultural differences, particularly those shaped by individualistic versus collectivist values, shape parenting practices. This is observed as marked contrasts in caregiving across the Global South versus the North. While mothers from the Global North used more verbal cues and responses to engage with their children, those from the Global South were more likely to use physical gestures such as stroking, patting or kissing as forms of responsive care. This is potentially attributable to cultural beliefs in certain Global South regions that deemed parent-initiated conversations with a child who is ‘too young to understand’ as “foolish” [145]. These observations call for attention to tailoring interventions based on cultural norms, beliefs, and value systems, to make them more acceptable to the populations for whom they are designed.

Additionally, urban caregivers generally exhibited higher sensitivity, perhaps due to availability of more resources and fewer distractions or chores. Similarly, studies from low-income settings such as urban slums in Rio de Janeiro, Brazil, Makassar, and Indonesia reported low levels of caregiver sensitivity, potentially attributable to the myriad of stressors associated with poverty [191,192]. The relative success of structural interventions such as income supplementation and community support suggests that these measures could lower financial stressors, which could potentially translate to improved responsive caregiving among the most vulnerable populations.

Parenting behaviour emerges from the interplay of three key domains: caregivers’ personal resources, child factors, and broader contextual factors [7,8]. In line with this, our review found that caregiver factors such as poor physical or mental health are related to lower levels of responsiveness. Furthermore, child factors such as preterm birth or developmental delays, which are likely to elicit subtler socio-emotional cues, are also likely to impact the caregivers’ ability to provide responsive caregiving. Simultaneously, factors such as poverty, particularly when combined with limited social support, also adversely impact responsive caregiving. Therefore, while most studies frame responsive caregiving as a function of static demographic attributes, in reality, it is a dynamic, relational process embedded in individual psychological capacities such as noticing and accurately interpreting children’s cues, regulating emotions, and maintaining the intention to respond. For instance, adolescent mothers were found to be less responsive compared to older mothers; however, this difference may also be influenced by other related factors to maternal age, such as potentially greater stigma, less caregiving experience, or limited social support. Future research should move beyond describing correlates to examining how and why these factors may influence caregiver-child interaction quality, using theory-informed and mechanism-based approaches to unpack the dynamic interplay of context, caregiver capacity, and child needs.

### Association of responsive caregiving with child development

Studies overwhelmingly support the positive association of responsive caregiving on a child neurodevelopment, with language development being the most commonly studied. High levels of maternal responsiveness were also associated with better cognitive and literacy skills, including for children born preterm. Evidence from randomized trials in this review, and across the global literature also support that improvements in responsive caregiving are positively associated with children’s development [193]. However, most of the evidence in this review was based on cross-sectional studies, so the direction of effect cannot be confirmed. Therefore, additional research is needed to strengthen and clarify these associations.

### Limitations

By including peer-reviewed articles in English, this review may have excluded relevant studies in other languages or non-peer-reviewed sources. However, this was done to ensure that the authors were able to correctly interpret the findings of the included papers. The broad contexts, populations, and settings in which responsive caregiving has been studied, along with the different frameworks used to conceptualize it, made it challenging to comprehensively incorporate all these concepts into the search strategy while maintaining the feasibility of conducting the scoping review. Therefore, we focused on screening articles that included terms and concepts of responsive caregiving within the title and/or abstract. Articles that may have alluded to responsive caregiving only in the full-text without explicitly mentioning them in the title or abstract, or described concepts of responsive caregiving that are different from the terminology typically used within the nurturing care framework, may have been missed. While being cognizant of this limitation, we followed the search strategy described in Supplementary file 2 to complete this review. Going forward, it is crucial that the field establishes a clear operational definition of responsive caregiving and delineates its specific measurement domains [27,194]. This will not only aid in developing and validating contextualized tools to measure responsive caregiving across diverse global settings and populations, but will also help standardize measurements and reporting guidelines to allow comparisons of responsive caregiving across populations in future reviews.

The following limitations were noted for the studies included in this review: (i) most studies involved small and homogenous sample sizes (63 % with *n* < 100; lowest *n* = 7 mother-child dyads), thereby limiting the generalizability of the findings. Findings based on single, context-specific studies should therefore be interpreted cautiously, viewed as indicators of gaps in the literature rather than as conclusive evidence; (ii) several studies assessed caregiver-child interactions in laboratory settings using structured tasks, thus challenging their ecological validity. It is also worth noting the low frequency and duration of the interactions, further warranting the need to observe interactions in the natural context for reasonably long periods of time; (iii) lack of consistency in the definitions, frameworks, and measurement tools used to assess responsive caregiving made it difficult to directly compare the results across studies. Even when the same responsive caregiving domain was measured (such as maternal responsivity), the methodologies and tools varied significantly (e.g., HOME, NCATS). This heterogeneity precluded the possibility of a meta-analysis. Consequently, we conducted a scoping review, presenting a narrative synthesis to map and summarize the available evidence; (iv) validity of some of the tools used can be questioned since they were used in contexts, cultures, developmental stages, and environmental settings that were different from the settings in which these tools were initially validated. Finally, (v) the limited focus on mechanisms and pathways such as stressors, facilitators, and barriers that underlie differences in caregiving behaviours and child development.

### Recommendations

#### 1 Expand research in the global South

There is a pressing need for more studies based in the Global South, especially since there are clear differences in responsive caregiving between the Global South versus the North. Furthermore, a more nuanced understanding of the commonalities and differences in responsive caregiving across diverse Global South settings is also warranted, to help develop culturally nuanced and contextualized interventions, policies, and practices.

#### 2 Broaden caregiver representation beyond mothers

Most studies focused on mother-child interactions to assess responsive caregiving; however, future studies should include a broader range of caregivers, including fathers, grandparents, siblings, and extended family members, to examine their unique contributions and experiences in providing nurturing care. This is particularly important in societies in which caregiving is a shared responsibility. Similarly, this review demonstrated that paternal involvement positively impacts a broad range of children’s outcomes. Therefore, we strongly advocate for the inclusion of fathers as essential partners in providing responsive care by providing them with the necessary support, education, resources, and role-models.

#### 3 Need for context-specific, validated measurement tools

A crucial prerequisite for advancing the field and achieving consistency in reporting metrics related to responsive caregiving is to develop context-specific validated measurement tools based on a clear and universally applicable definition of responsive caregiving. Qualitative methods are an appropriate approach for studying the cultural nuances of responsive caregiving in different contexts; therefore, our review highlights the need for more mixed-methods approaches to be used in future research.

#### 4 Target and refine interventions based on population and contexts across multiple levels of enquiry

Our review highlights the importance of using a multilevel approach when considering factors influencing responsive caregiving – beginning with the caregiver-child dyad, but extending to neighbourhoods and communities, and broader policy interventions at the national and global levels. First, we need more research to develop a broader view of the factors influencing responsive caregiving in different contexts that extend to pathways and mechanisms such as stressors, facilitators, barriers, to name a few, which will then help inform the design of multi-level and multicomponent interventions that can create synergistic benefits by facilitating responsive caregiving not only within the home by immediate family members, but also in neighbourhoods, schools, and other early childhood care and education settings by teachers and other care providers. Future research is also needed to explore how responsive caregiving varies both within and across cultural contexts, to better inform culturally sensitive interventions. By integrating efforts across multiple levels, interventions can promote nurturing environments that more comprehensively and holistically support children’s development.

## 6 Conclusion

This scoping review highlights responsive caregiving and their determinants across different contexts and populations worldwide, and identifies variations based on caregiver, child, socio-economic, and cultural factors. These findings suggest that caregivers adapt their responsive caregiving in context to developmental and contextual changes. Importantly, responsive caregiving is significantly associated with children’s language, cognitive, and motor development. However, more research is needed to better understand the nuances of responsive caregiving in underrepresented populations in the Global South, and to validate their associations with children’s development. Policymakers and practitioners must consider the socio-cultural contexts along with pathways and mechanisms that influence caregiving to design effective, inclusive interventions that optimize responsive caregiving and support child development worldwide.

## Supplementary Material

Supplementary data to this article can be found online at https://doi.org/10.1016/j.earlhumdev.2025.106424.

Supplementary Material

## Figures and Tables

**Table 1:** Characteristics of studies included in this review (*N* = 138).

Category		No. of studies
***Study year***	1982–1992	7
	1993–2002	18
	2003–2012	47
	2013–2024	66
***Regions*** ^a,b^	Americas	81
	Europe	42
	Africa	12
	Western Pacific	9
	Southeast Asia	6
	Eastern Mediterranean	2
***Sample size***	Less than 100	87
	100 to 500	45
	*>*500	6
***Type of studies***	**Quantitative**	133
	Cross-sectional	80
	Longitudinal or cohort	42
	Randomized controlled trial	10
	Case-control	1
	**Qualitative**	1
	**Mixed methods**	4
***Research question 1:***		
**Responsive caregiving in relation to caregiver factors**		
*Caregiver physical and mental health*		28
*Caregiver demographics*		15
*Caregiver relation to child (mothers, fathers, biological, adopted,* *naturally conceived, medical assistance for conception, daycare* *providers)*		13
*Caregiver feeding practices*		11
**Responsive caregiving in relation to child factors**		
*Child developmental status*		27
*Child demographics*		20
*Child health*		3
**Responsive caregiving in relation to context**		
*Cultural/location differences*		16
*Socio-economic status*		8
***Research question 2:***		38
**Association between responsive caregiving and child development**		

a Nine studies were done in more than one country

b based on the WHO classification of regions.

**Table 2:** Characteristics, measurement tools, constructs, and quality of each study included in the scoping review.

Authors	Year	Sample size*	Study type#	Study design	Country	Caregiver factors	Child factors	Cultural/location	Socio-economic	Child development	Measurement tool	Construct measured (as indicated in the study)	Quality
Abebe et al.	2017	2	1	Cross-sectional Cluster-randomized	Ethiopia	X	X				Videotaped mother-child interactions; Modified coding scheme adapted from Moore et al.	Responsiveness	Medium
Aboud et al.	2011	2	1	controlled trial	Bangladesh	X				X	Videotaped mother-child interactions coded Videotaped mother-child interactions;	Responsiveness	High
Agostini et al.	2014	2	1	Cohort	Italy		X				Global ratings scale by Murray et al.	Sensitivity	High
Alsarhi et al.	2020	1	1	Cross-sectional	Yemen	X		X			Ainsworth sensitivity scale	Sensitivity	Medium
Alvarez	2004	1	1	Cohort	Denmark		X				24-h behaviour diary; Coding based on Crockenberg and Smith scale	Responsiveness	High
											
Anke et al.	2019	1	1	Cross-sectional	Norway	X					Videotaped mother-child interactions; Parent-Child Early Relational Assessment (PCERA)	Positive affect, sensitivity, responsiveness	High
Armony-Sivan et al.	2010	1	1	Cross-sectional	USA		X				Videotaped mother-child interactions; Nursing Child Assessment Feeding Scale (NCAFS)	Sensitivity to cues, response to distress	High
Asanjarani et al.	2021	1	3	Mixed	Iran			X			Videotaped mother-child interactions; Ainsworth sensitivity scale; Qualitative observations	Sensitivity	Medium
Baptista et al.	2019	1	1	Cross-sectional	Portugal Spain		X				Videotaped mother-child interactions; Ainsworth sensitivity scale	Sensitivity	High
											
Barratt et al.	1996	1	1	Cohort	USA		X				Videotaped mother-child interactions; Home Observation for Measurement of the Environment (HOME) Inventory	Emotional and verbal responsivity	High
Beebe et al.	2018	1	1	Cross-sectional	USA		X				Videotaped mother-child interactions	Contingency	High
Beer et al.	2013	1	1	Cross-sectional	UK		X				Nursing Child Assessment Teaching Scale (NCATS); HOME	Sensitivity to cues, response to distress, responsivity	High
Binda et al.	2019	2	1	Cross-sectional	Chile	X					Child-Adult Relationship Experimental Index (CARE-Index)	Sensitivity	High
Black et al.	1994	1	1	Randomized controlled trial	USA	X				X	HOME	Emotional and verbal responsivity	High
Boechler et al.	2003	2	1	Cross-sectional	Canada	X					NCATS	Sensitivity to cues, response to distress	Medium
Bornstein et al.	1992	1	1	Cross-sectional	USA France Japan			X			Videotaped mother-child interactions coded	Responsiveness	High
Bornstein et al.-1	2008	1	1	Cohort	USA		X				Videotaped mother-child interactions; Event-based coding	Responsiveness	High
Bornstein et al.-2	2008	2	1	Cross-sectional	Argentina Italy USA		X	X			Videotaped mother-child interactions; Emotional Availability Scales: Infancy to Early Childhood Version	Sensitiveness, responsiveness	High
Bornstein et al.	2012	2	1	Cross-sectional	Japan USA			X			Videotaped mother-child interactions coded	Contingency	High
Bornstein et al.	2021	2	1	Case-control	USA	X					Videotaped mother-child interactions coded	Contingency	High
Broesch et al.	2016	1	1	Cross-sectional	Fiji, Kenya, USA			X			Videotaped mother-child interactions coded	Contingent responsiveness	High
Broom	1998	2	1	Cohort	USA	X					NCATS	Sensitivity to cues, response to distress	Medium
Brooks-Gunn & Lewis	1984	2	1	Cross-sectional	USA		X				Videotaped mother-child interactions coded	Responsivity	High
Chaudhary et al.	2023	1	1	Cross-sectional	Republic of Congo			X			Videotaped caregiver-child interactions	Sensitive responsiveness	High
Cheng et al.	2018	1	1	Cohort	China					X	Videotaped mother-child interactions; Maternal behaviour Q-sort (MBQS)	Sensitivity	High
Chiarello et al.	2006	1	1	Cross-sectional	USA	X				X	Videotaped caregiver-child interactions; Maternal Behaviour Rating Scale	Responsiveness	High
Chico et al.	2014	1	1	Cross-sectional	Canada	X					Videotaped mother-child interactions; Ainsworth Maternal Sensitivity Scales	Sensitivity	High
Cooper et al.	2009	2	1	Randomized controlled trial	South Africa	X					Videotaped mother-child interactions	Sensitivity	High
Corapci et al.	2006	2	1	Cross-sectional	Costa Rica		X				Videotaped mother-child interactions; Parent-Child Interaction System (PARCHISY); Early Childhood version of the HOME (EC-HOME)	Positive affect, responsivity, verbalization	High
Dalen et al.	2020	3	1	Cross-sectional	Norway	X					Videotaped mother-child interactions; Global rating system by NICHD Study of Early Child Care and Youth Development	Sensitivity, positive regard	High
Dave et al.	2018	1	1	Cohort	USA					X	Videotaped mother-child interactions coded	Responsive utterance	Medium
Dearden et al.	2009	1	1	Cross-sectional	Vietnam	X					Mother-child interactions; Coding scheme adapted from Bentley et al.	Positive verbalization	Medium
Delehanty et al.	2024	2	1	Cross-sectional	USA					X	Videotaped caregiver-child interactions; Coded adapted from Cress et al., Haebig et al., Tamis-LeMonda et al.	Verbal responsiveness	High
Doussard-Roosevelt et al.	2003	1	1	Cross-sectional	USA		X				Videotaped mother-child interactions coded	Sensitivity	High
Eiden et al.	2011	2	1	Cross-sectional	USA	X					Videotaped caregiver-child interactions; Mother-Infant Feeding Scale; PCERA	Sensitivity, positive involvement	High
Endevelt-Shapira et al.	2024	1	1	Cohort	USA					X	Coding Interactive Behaviour (CIB) system	Sensitivity	High
Engelke et al.	1992	2	1	Cross-sectional	USA	X					HOME	Emotional and verbal responsivity	Medium
Erickson et al.	2018	2	1	Cross-sectional	USA		X			X	Videotaped mother-child interactions; C-CARES	Responsiveness, positive affect	High
Esposito et al.	2017	1	1	Cross-sectional	USA	X					Videotaped mother-child interactions; Coding schema adapted from Bornstein	Responsiveness	High
Feeley et al.	2011	1	1	Cross-sectional	Canada	X					Emotional availability scale; Videotaped mother-child interactions	Sensitivity	Medium
Fink et al.	2024	2	1	Cohort	England					X	Still Face paradigm	Sensitivity measured at baseline & reunion episodes	High
Flippin et al.	2015	1	1	Cross-sectional	USA	X				X	Videotaped caregiver-child interactions; Coding schema adapted from Yoder, Fey, Thompson, McDuffie, and Lieberman	Verbal responsiveness	High
Flykt et al.	2010	1	1	Cohort	Finland	X	X				Videotaped caregiver-child interactions; CARE index	Sensitivity	High
Flynn & Masur	2007	1	1	Cohort	USA		X			X	Videotaped mother-child interactions coded	Responsiveness	High
Fourment et al.	2018	1	3	Mixed	Peru	X		X			Ainsworth sensitivity scale; Videotaped mother-child interactions	Sensitivity	Medium
Garcia et al.	1986	1	1	Cohort	USA	X					HOME; Videotaped mother-child interactions	Emotional and verbal responsivity	Medium
Gerstein et al.	2019	1	1	Cohort	USA	X					Videotaped mother-child interactions; Parent Child Interaction Rating Scale	Sensitivity	High
Giardino et al.	2008	1	1	Cross-sectional	Canada	X					Videotaped mother-child interactions coded	Responsiveness	High
Girolametto et al.	2002	1	1	Cross-sectional	Italy Canada			X		X	Videotaped mother-child interactions coded	Responsiveness	High
Gladstone et al.	2018	2	2	Qualitative	Malawi			X	X		Observations, In-depth interviews, focus groups, Participatory Research focus groups	Responsive caregiving	High
Golombok et al.	2006	2	1	Cross-sectional	UK	X					Standardized interviews coded	Sensitive responding	High
Harel-Gadassi et al.	2020	2	1	Cohort	Israel		X				Videotaped mother-child interactions; CIB	Sensitivity	High
Holditch-Davis et al.	2003	1	1	Cross-sectional	USA		X				Videotaped mother-child interactions; HOME	Positive affect, responsivity	Medium
Hurtado-Mazeyra et al.	2022	3	1	Cohort	Peru					X	Caregiver interview	Sensitivity	High
Hwa-Froelich et al.	2008	1	1	Cross-sectional	USA	X					Videotaped mother-child interactions; Clarke-Stewart Rating Scale	Sensitivity	High
Imhof et al.	2023	1	1	Randomized controlled trial	USA					X	Videotaped caregiver-child interactions	Responsive caregiving	High
Jahromi et al.	2004	2	1	Cohort	USA		X				Videotaped mother-child interactions coded	Responsiveness	High
Johnson and Lobo	2001	1	1	Cross-sectional	USA	X					NCATS	Sensitivity to cues, response to distress	High
Kagawa et al.	2017	3	3	Mixed	Mexico	X					HOME; Semi-structured qualitative interviews	Responsivity	High
Kärtner et al.	2008	2	1	Cross-sectional	Germany USA India Cameroon China			X			Videotaped mother-child interactions; Event-coding approach	Contingent responsiveness	High
Kärtner et al.	2010	1	1	Cohort	Germany, Cameroon			X			Videotaped mother-child interactions; Event-coding approach	Contingent responsiveness	High
Keller et al.	2008	1	1	Cohort	Germany, Cameroon		X	X			Videotaped mother-child interactions coded	Contingent response	High
Konijnenberg et al.	2016	1	1	Cross-sectional	Norway	X					Videotaped mother-child interactions; Assessment by National Institute of Child Health and Human Development (NICHD)	Sensitivity/responsiveness, positive affect	High
Koshy et al.	2021	2	1	Cohort	India					X	HOME	Emotional and verbal responsivity	High
Laing et al.	2010	1	1	Cross-sectional	USA		X				Videotaped mother-child interactions; NICHD Qualitative Scales of Observational Ratings	Responsivity	High
Landau et al.	2009	1	1	Cross-sectional	Israel		X				Videotaped mother-child interactions coded	Responsivity	High
Landry et al.	2003	2	1	Cohort	USA		X			X	Videotaped mother-child interactions coded	Responsiveness	High
Laude	1999	2	1	Cross-sectional	Costa Rica					X	NCATS; HOME	Sensitivity to cues, response to distress, emotional and verbal responsivity	High
Lavelli et al.	1998	1	1	Cohort	Italy	X					Videotaped mother-child interactions coded	Responsive vocalization	High
Lee and Ha	2023	1	1	Cohort	South Korea		X				LENA system	Verbal responsiveness	High
Levickis et al.	2014	2	1	Cohort	Australia					X	Videotaped mother-child interactions coded	Responsive behaviour	High
Lewis et al.	2009	2	1	Cross-sectional	London	X					Ainsworth sensitivity scale	Sensitivity	High
Lorang et al.	2018	1	1	Cross-sectional	USA		X				Videotaped mother-child interactions coded	Responsivity	High
Losier et al.	2020	1	1	Cross-sectional	Canada		X				Mini-Maternal Behaviour Q-Sort (mini-MBQS-V)	Sensitivity	High
Maguire et al.	2016	1	1	Cross-sectional	USA	X					Videotaped mother-child interactions; Nursing Child Assessment Satellite-Training Scale (NCAST-Feeding)	Sensitivity to cues, response to distress, contingency	High
McDuffie et al.	2010	1	1	Cohort	USA					X	Videotaped caregiver-child interactions coded	Verbal responsiveness	High
McGrath et al.	1998	2	1	Cross-sectional	USA					X	HOME	Emotional and verbal responsivity	High
Mesman et al.	2018	1	3	Cross-sectional	Kenya	X		X			Ainsworth sensitivity scale	Sensitivity	High
Minde et al.	1988	2	1	Cohort	Canada		X				Videotaped mother-child interactions coded	Contingency	High
Moore et al.	2006	1	1	Cross-sectional	Bangladesh	X					Videotaped parent-child interactions coded	Responsiveness	High
Murray et al.	1996	2	1	Cohort	England	X					Videotaped mother-child interactions	Sensitivity	High
Murray et al.	2003	2	1	Randomized controlled trial	USA	X					Videotaped mother-child interactions; Global ratings scale by Murray et al.	Sensitivity	High
Murray et al.	2005	1	1	Cross-sectional	UK	X					Interviews adapted from Quinton and Rutter	Sensitivity	High
Murray et al.	2008	2	1	Cohort	London		X				Videotaped mother-child interactions	Responsiveness	High
Murray et al.	2016	1	1	Randomized controlled trial	South Africa					X	Videotaped mother–child interactions; Coding scheme by Cooper et al.	Sensitivity	High
Mutoro et al.	2019	1	1	Cross-sectional	Kenya	X					Caregiver-child interactions coded	Responsive feeding	Medium
Neri et al.	2017	1	1	Cross-sectional	Italy	X					Videotaped mother–child interactions; CARE-Index	Sensitivity	High
Neumann et al.	2020	2	1	Cross-sectional	USA			X			Videotaped mother-child interactions; Ratings for sensitivity/responsiveness and synchrony/reciprocity	Sensitivity/responsiveness	Medium
Nicol-Harper et al.	2007	1	1	Cross-sectional	UK	X					Videotaped mother–child interactions; Coding adapted from Stein et al.	Sensitive responsivity	High
Nicolson et al.	2013	1	1	Randomized controlled trial	Australia	X					Videotaped mother-child interactions; Emotional Availability Scales (EAS) (fourth edition)	Sensitivity	Medium
Olson and Masur	2015	1	1	Cross-sectional	USA					X	Videotaped mother-child interactions coded	Responsive labelling	High
Oosterom et al.	2020	2	1	Cohort	The Netherlands		X				Videotaped mother-child interactions; CIB	Sensitivity	High
Ostfeld et al.	2000	1	1	Cohort	USA		X				Modified Beckwith Mother-Infant Behaviour Checklist; HOME	Responsiveness, emotional and verbal responsivity	Medium
Pajulo et al.	2011	1	1	Cohort	Finland	X				X	Videotaped mother-child interactions; CARE-Index	Sensitivity	Medium
Paris et al.	2009	1	1	Cross-sectional	USA	X					Videotaped mother-child interactions; CIB	Sensitivity and responsiveness	High
Pearson et al.	2011	3	1	Cohort	UK					X	Videotaped mother-child interactions; Thorpe Interaction Measure	Sensitivity	High
Perea-Velasco et al.	2023	1	1	Cohort	Spain		X				CITMI-R: early mother–child interaction coding system, revised edition)	Sensitivity	High
Perez et al.	2005	1	1	Randomized controlled trial	South Africa	X					Videotaped mother–child interactions; Parent/Caregiver involvement scale	Responsiveness	High
Pino	2000	1	1	Cross-sectional	Italy		X				Videotaped mother-child interactions coded	Positive verbalization	High
Radesky et al.	2015	2	1	Cross-sectional	USA	X					Videotaped mother-child interactions; BATMAN (Bob and Tom’s Method of Assessing Nutrition)	Contingent responsiveness	High
Rafferty et al.	2011	3	1	Cohort	USA	X				X	Videotaped mother-child interactions; NICHD; HOME-Short Form	Supportiveness, responsivity	High
Rahma et al.	2021	1	1	Cross-sectional	Indonesia	X			X		Videotaped mother–child interactions; Ainsworth sensitivity scale	Sensitivity	High
Räihä et al.	2002	1	1	Cross-sectional	Finland	X					Videotaped caregiver-child interactions; PCERA	Sensitivity, responsivity	High
Ribe al	2018	2	1	Cohort	Tanzania					X	HOME	Emotional and verbal responsivity	High
Ribeiro-Accioly et al.	2021	1	1	Cross-sectional	Brazil				X		Videotaped mother-child interactions; Ainsworth sensitivity scale	Sensitivity	High
Roach et al.	1998	1	1	Cross-sectional	USA		X				Videotaped mother-child interactions coded	Contingent responsiveness	High
Roberts et al.	1999	1	1	Cohort	USA					X	HOME	Emotional and verbal responsivity	Medium
Roberts et al.	2002	1	1	Cohort	USA					X	HOME	Emotional and verbal responsivity	Medium
Scherer et al.	2019	3	1	Cross-sectional	Pakistan	X			X	X	Observation of Mother- Child Interaction (OMCI); HOME	Responsivity, positive affect, sensitivity	High
Schermann-Eizirik et al.	1997	2	1	Cohort	Sweden		X				Videotaped mother-child interactions; Observational protocols based on Bohlin et al., Eizirik et al.	Sensitivity	Medium
Schiffman et al.	2003	2	1	Cross-sectional	USA				X		Nursing Child Assessment Satellite Training (NCAST) Teaching Scale	Sensitivity to cues, response to distress	High
Shaw et al.	2006	1	1	Cross-sectional	USA	X	X				Videotaped mother-child interactions coded	Responsivity	High
Siller et al.	2013	1	1	Randomized controlled trial	USA					X	Videotaped mother-child interactions coded	Responsive verbal behaviours	High
Smith et al.	2017	2	1	Cross-sectional	Australia					X	Videotaped mother-child interactions coded	Responsiveness	High
Sonobe et al.	2016	1	1	Cohort	Japan	X					Videotaped mother-child interactions; NCATS	Sensitivity to cues, response to distress	Medium
Soukup-Ascencao et al.	2016	1	1	Cross-sectional	UK		X				Videotaped caregiver-child interactions; Social Interaction Measure for Parents and Infants (SIM-PI)	Sensitive responsiveness	Medium
Sterling et al.	2013	1	1	Cross-sectional	USA	X	X				Videotaped mother-child interactions; Coded system adapted from Landry et al.	Responsivity	High
Sterling and Warren	2014	1	1	Cross-sectional	USA		X				Videotaped mother-child interactions; Coding system adapted from Landry et al.	Responsivity	Medium
Sterling and Warren	2018	1	1	Cross-sectional	USA		X				Videotaped caregiver-child interactions coded	Responsivity	Medium
Stith et al.	1984	1	1	Cross-sectional	USA	X				X	Videotaped mother-child interactions; Yarrow, Rubenstein, and Pedersen’s infant environment observational scale	Contingent responsiveness, positive affect	Medium
Stuckey et al.	1982	1	1	Cross-sectional	USA	X	X				Videotaped caregiver-child interactions; Revision of Family Observation Manual	Positive affect	High
Suwalsky et al.	2012	1	1	Cross-sectional	USA	X	X				Videotaped mother-child interactions coded	Contingent response	High
Tamis-LeMonda et al.	2001	1	1	Cohort	USA					X	Videotaped caregiver-child interactions; Coding based on Bornstein and Tamis-LeMonda et al.	Responsiveness	High
Tamis-LeMonda et al.	2004	2	1	Cohort	USA	X	X		X	X	Videotaped caregiver-child interactions; Scales adapted from the NICHD Study of Early Child Care’s Three Box scales	Sensitivity, positive regard	High
Taylor et al.	2008	2	1	Cohort	USA		X			X	Mother-child interactions coded	Responsiveness	High
Till et al.	2019	1	1	Cross-sectional	Costa Rica			X			NCATS	Sensitivity to cues, response to distress	High
Vaccaro et al.	2021	1	1	Cross-sectional	USA		X		X		Videotaped mother-child interactions; NICHD coding system for maternal sensitivity	Sensitivity	High
van Vliet et al.	2022	2	1	Cross-sectional	The Netherlands	X					Videotaped mother-child interactions; Ainsworth sensitivity scale	Sensitivity	High
Vazir et al.	2013	2	1	Randomized controlled trial	India					X	Videotaped parent-child interactions	Responsive feeding	Medium
Vilaseca et al.	2020	1	1	Cross-sectional	Spain	X	X				Videotaped caregiver-child interactions; PICCOLO	Responsiveness	Medium
Vinall et al.	2013	2	1	Cross-sectional	Canada					X	Videotaped parent-child interactions; Emotional Availability Scale IV	Sensitivity	High
Wade et al.	2008	2	1	Cross-sectional	USA		X				Videotaped caregiver-child interaction; Coding system used by Landry et al.	Responsiveness	High
Wallace et al.	1998	1	1	Cross-sectional	USA				X		Videotaped mother-child interactions; HOME; NCATS	Sensitivity, responsiveness	Medium
Walton et al.	2015	1	1	Cross-sectional	USA					X	Videotaped mother-child interactions coded	Responsiveness	High
West and Iverson	2021	1	1	Cohort	USA		X				Videotaped caregiver-child interactions coded	Contingent verbal response	High
Wheeler et al.	2007	1	1	Cross-sectional	USA		X				Videotaped caregiver-child interactions; Maternal Rating Scale	Sensitivity	High
Wheeler et al.	2010	1	1	Cross-sectional	USA		X				Modified version of the transparent box episode	Responsivity	High
Whitfield et al.	2019	1	1	Cross-sectional	Canada	X					Videotaped caregiver-child interactions; NCAST Caregiver/Parent-Child Interaction Feeding Scale	Sensitivity to cues, response to distress	Medium

* **sample size** - 1 = Less than 100; 2 = 100 to 500; 3 ≥500

# **study type -** 1 = quantitative; 2 = qualitative; 3 = mixed methods.

## Data Availability

All relevant data are available within the paper and supplementary files.
